# Information Easiness Affects Non-experts’ Evaluation of Scientific Claims About Which They Hold Prior Beliefs

**DOI:** 10.3389/fpsyg.2021.678313

**Published:** 2021-08-27

**Authors:** Lisa Scharrer, Rainer Bromme, Marc Stadtler

**Affiliations:** ^1^Department of Educational Science, Ruhr-University Bochum, Bochum, Germany; ^2^Department of Psychology, University of Muenster, Muenster, Germany

**Keywords:** public understanding of science, knowledge evaluation, science comprehension, easiness, prior beliefs, belief consistency

## Abstract

Usually, non-experts do not possess sufficient deep-level knowledge to make fully informed evaluations of scientific claims. Instead, they depend on pertinent experts for support. However, previous research has shown that the easiness by which textual information on a scientific issue can be understood seduces non-experts into overlooking their evaluative limitations. The present study examined whether text easiness affects non-experts’ evaluation of scientific claims even if they possess prior beliefs about the accuracy of these claims. Undergraduates who strongly believed that climate change is anthropogenic read argumentative texts that were either easy or difficult to understand and that supported a claim either consistent or inconsistent with their beliefs. Results are consistent with the hypothesis that text easiness affects non-experts’ judgment of scientific claims about which they hold prior beliefs—but only when these claims are in accordance with their beliefs. It seems that both text difficulty and belief inconsistency remind non-experts of their own limitations.

## Introduction

The climate crisis or the COVID-19 pandemic are prime examples of scientific issues whose immediate relevance to the broad public leads many to consider scientific knowledge in their behavioral choices. Because of its strong impact, it is vital that people critically evaluate the scientific information they encounter in order to distinguish reliable knowledge claims from misinformation in the shape of “alternative facts,” pseudoscience, and conspiracy theories—especially if information is published online and thus frequently not subject to any editorial control (Barzilai and Chinn, 2020; Macedo-Rouet et al., 2020). Failure to make this distinction can lead to harmful or, in cases such as COVID-19, possibly even lethal decisions (Dhanani and Franz, 2020).

However, critically evaluating scientific information is a difficult task. It requires broad background knowledge and experience that is usually to be found only in trained experts in the field. Their specific training and experience in a particular domain distinguishes experts from non-experts (Bromme et al., 2001). Non-experts seeking to base their decision making on reliable scientific knowledge therefore face the paradoxical challenge of needing to evaluate scientific information despite usually not having a “domain insider’s” sufficient level of topic knowledge to make fully informed judgments. To resolve this paradox, non-experts need to act as “competent outsiders” (Feinstein, 2011; Tabak, 2015; Duncan et al., 2018), drawing on skills that allow them to evaluate scientific information indirectly. Such indirect evaluation can be achieved by identifying pertinent experts or expert sources on whose judgment non-experts can rely and to whom they can outsource the evaluative task (Bromme et al., 2018; Collins et al., 2018; Keren, 2018).

A problem arises when non-experts are unaware of this dependency, and ultimately of their outsider status. Studies on the easiness effect of science popularization have shown that the easiness by which textual information on a scientific issue can be understood tends to increase persuasiveness and seduces non-experts into overestimating their own evaluative capabilities (e.g., Scharrer et al., 2012, 2017). The easiness effect therefore increases non-experts’ vulnerability to easily understandable misinformation.

The effect is of practical relevance, because non-experts seeking to inform themselves about scientific issues are often confronted with easily understandable accounts. Science journalists specifically design popularized scientific reports to inform non-expert audiences. To make knowledge accessible and understandable to their target audience, these reports are usually simplified by translating technical terms into more familiar language or by omitting complex details (e.g., Singer, 1990; Goldman and Bisanz, 2002; Hijmans et al., 2003; Tabak, 2015, 2018).

Apart from these more traditional means of science communication, non-experts also encounter simplified depictions on social media (Hargittai et al., 2018), where the production of easy, straightforward messages may be motivated by space restrictions or the desire to attract attention. Finally, simplified accounts of scientific issues may also be communicated as part of political messages, particularly by populist politicians who attempt to appeal to voters with easily understandable propositions and solutions (Bischof and Senninger, 2018).

Up to now, investigations of the easiness effect have focused on situations in which people did not possess any relevant prior knowledge or beliefs about the scientific claims (e.g., Scharrer et al., 2012, 2013, 2019). Against this background, the present study addresses the question whether the easiness of scientific information affects non-experts’ evaluation of science-based claims when they hold prior beliefs about the claims in question. This question is not trivial, given that scientific issues of societal and individual importance are often the subject of broad public debate. Consequently, it is not rare for non-experts to bring previously formed beliefs to the table.

### The Easiness Effect of Science Popularization

Studies on processing fluency have shown that in many contexts, the subjective experience of ease of performing a mental task positively affects people’s judgment of truth and confidence (e.g., Reber and Schwarz, 1999; Schwarz, 2004; Alter and Oppenheimer, 2009). Further research has shown that the ease of processing also influences non-experts’ perceived ability to evaluate scientific information (e.g., Scharrer et al., 2012, 2017). In a study by Scharrer et al. (2012), non-experts were confronted with argumentative texts supporting causal claims about different medical issues. Texts varied in terms of the easiness by which they could be processed, operationalized by either including a large number of technical terms (difficult condition) or by translating these terms into words familiar to non-experts (easy condition). It is important to note that both versions addressed the same complex scientific issue, so that a reliable judgment would obviously require scientific expertise. After reading, participants agreed more with claims supported by easy rather than difficult-to-understand arguments. In addition, they expressed more confidence in their own claim judgment and conversely a reduced desire to consult an expert for judgment support. It appears that the easy understanding of scientific information seduced participants into believing that their abilities were sufficient not only to comprehend but also to reliably judge the science information they had encountered. This, however, is a false conclusion, because the ease by which information can be understood is usually not indicative of the easiness (or lack of complexity) of the scientific topic it addresses. Thus, even though non-experts might have understood a snippet of topic information, this does not, of course, equip them with sufficient deep-level background knowledge to adequately evaluate its validity.

Another study has shown that this easiness effect does not just occur with artificial text materials specifically designed for experimental purposes but also with authentic journalistic reports. Non-experts are more inclined to confidently agree with reports designed for lay audiences than with more difficult reports targeted at expert readers (Scharrer et al., 2017).

The easiness effect has been shown to be robust against variations of scientific discipline/topic and contextual factors (Scharrer et al., 2013, 2014, 2019; Thon and Jucks, 2017; Bullock et al., 2019). The effect can be reduced if non-experts are made aware of the controversial nature of the claim in question (Scharrer et al., 2013) or if non-experts are warned that the topic at hand is actually very complex (Scharrer et al., 2014). Nonetheless, such measures do not prevent the easiness effect completely. In addition, the effect even occurs if information is presented by an obviously non-credible source. Apparently, and also alarmingly, sources can compensate for their lack of credibility by presenting easily understandable information (Scharrer et al., 2019).

Many of these past studies on the influence of information easiness intentionally used claims of a fictitious nature to keep participants’ prior knowledge and beliefs as low as possible (Scharrer et al., 2012, 2013, 2014, 2019). Other studies used authentic claims, but did not control for participants’ prior beliefs (Scharrer et al., 2017; Thon and Jucks, 2017; Bullock et al., 2019).

### Prior Beliefs Affect the Evaluation of Scientific Claims

A large body of research from educational, social, and cognitive psychology has shown that people’s prior beliefs strongly affect their evaluation of new information. They tend to evaluate information that is consistent with their prior beliefs more favorably, while discounting information that is inconsistent with them (e.g., Lord et al., 1979; Kunda, 1990; Tetlock, 1999; Hahn and Oaksford, 2007; Munro, 2010; McCrudden and Sparks, 2014; van Strien et al., 2016; Wolfe and Kurby, 2017; Hendriks et al., 2020). For example, Wolfe and Kurby (2017) asked participants to evaluate the soundness of arguments about different psychological topics. They found that participants rated those arguments whose claim was inconsistent with their prior beliefs to be less sound compared to arguments whose claims were belief-consistent.

This belief-consistency effect can arise as the result of a rational strategy. Readers may be hesitant to have their world view overthrown by a single piece of new information. Rather than dismissing their previous beliefs, they use these beliefs as a criterion by which to judge the plausibility of new incoming information (Richter and Schmid, 2010). This approach often makes sense from a competent outsider’s perspective if one’s prior belief is based on a rich body of reliable sources and their evidence.

However, the belief-consistency effect may also result from a motivational bias. Readers may seek to protect their beliefs, because these beliefs make them feel good about themselves or are part of their social identity (Kunda, 1990; Kahan, 2012; Sharon and Baram-Tsabari, 2020). The one-sided confirmation of belief-consistent and discounting of belief-inconsistent information as a result of motivated reasoning may prevent people from correcting false beliefs that are based on unreliable or outdated information and sources.

### The Interplay Between Prior Beliefs and Text Easiness

Goldberg and Carmichael (2017) investigated how text easiness affects claim evaluation when people possess prior beliefs about the issue at hand. They asked participants to read texts about policies that were either in accordance with or opposed to their prior political beliefs. In addition, the policies were written in either simple or complex language. The authors found that participants rated the belief-consistent policies more favorably if they were written in simple rather than complex language. In contrast, they discounted belief-inconsistent policies more strongly if the policies were written in simple compared to complex language. These findings might suggest that the easiness effect interacts with belief consistency in that belief consistency determines the direction of non-experts’ claim judgment (positive if belief-consistent, negative if belief-inconsistent), whereas text easiness determines the confidence in this judgment and therefore the strength of the positive/negative rating. However, it should be noted that Goldberg and Carmichael’s (2017) study focused on the evaluation of policies rather than scientific claims. Because policy claims are usually based on not only factual knowledge but also subjective values, it can be argued that this limits an evaluator’s dependence on experts compared to the situation with scientific claims. Hence, non-experts’ evaluation of policies does not necessarily follow the same rules as their evaluation of scientific claims. Moreover, the manipulation of language complexity affected the whole policy text, rendering it likely that it also influenced the effectiveness of the belief-consistency manipulation. Participants were probably better able to recognize whether the policy matched their beliefs in the simple language condition. As a result, it remains unclear whether the observed interplay is due to language affecting judgment differently depending on belief consistency or whether language influenced the extent that belief consistency could be recognized and considered in one’s judgment. Disentangling both explanations would require an independent variation of text easiness and belief consistency, ensuring that a claim’s belief consistency is recognized equally well regardless of the easiness of claim-supporting information.

If such an independence of both factors can be ensured, different ways are conceivable in which text easiness might affect the evaluation of scientific claims about which non-experts hold prior beliefs: first, it is possible that text easiness ceases to influence claim evaluation if non-experts can rely on their prior beliefs instead. Given previous findings on the strong influence of prior beliefs on claim assessment (e.g., Lord et al., 1979; Kunda, 1990; Munro, 2010), it is possible that non-experts ascribe so much weight to their prior beliefs as an evaluation criterion that there remains no room in which easiness might exert an influence.

Second, both prior beliefs and text easiness might affect non-experts’ claim evaluation, whereby belief-consistent information will generally be evaluated favorably and belief-inconsistent information will generally be devalued. Text easiness might determine the confidence of non-experts’ evaluation, so that easy belief-consistent information will be evaluated more favorably than easy belief-inconsistent information, whereas easy belief-inconsistent information will be rebutted more decisively than difficult belief-inconsistent information, analogous to the results by Goldberg and Carmichael (2017).

Finally, prior beliefs may moderate the easiness effect in that text easiness will affect the evaluation of only belief-consistent but not belief-inconsistent claims. If new information does not counter their previous beliefs, non-experts may be unshaken in their trust in their own evaluative capabilities. This trust will be further enhanced if they encounter information that is easy to understand, and they will be more likely to confidently rely on their judgment of this information. If information is belief-inconsistent, in contrast, the challenge of their previously held beliefs might give non-experts pause, alert them to their lack of topic knowledge that leaves them unable to resolve the conflict, and thereby create doubt about their own judgment capabilities. The induced skepticism about their own abilities might not even be alleviated by the easiness of belief-inconsistent information. This is tentatively suggested by findings from Scharrer et al. (2013), showing that the easiness effect is mitigated when non-experts become aware that the claim in question is controversially discussed among different sources. Perhaps such doubt is created by controversy not only between two external sources but also between a source and the non-expert’s own beliefs.

### The Present Study

The present study sought to advance our knowledge of how easiness affects non-experts’ evaluation of scientific claims when they possess prior beliefs about their accuracy. We tested how individuals’ reaction to such a situation corresponds to the three conceivable ways outlined above.

We confronted undergraduates holding strong prior beliefs about a scientific issue unrelated to their study subject with brief argumentative texts that supported a claim that was either consistent or inconsistent with these beliefs. In addition, the claim-supporting information was either easy or difficult to understand. After reading the text, we asked participants about their agreement with the proposed claim, and had them rate the credibility of the source providing the information. In addition, participants indicated their trust in their claim judgment based on their present knowledge, and their desire to consult an expert for judgment support.

The causes of climate change was chosen as the scientific issue in our stimulus materials, because this topic is of high societal relevance, and due to its broad public discussion, many people in Germany hold clear issue-related beliefs (DG COMM, 2019). In the current study, we focused only on individuals who believe that human behavior is the primary cause of climate change in order to prevent an overly complex study design that would introduce participants’ attitude regarding the cause of climate change as an additional factor. Moreover, survey data show that by far the largest group of Germans (49%) believe human behavior to be the primary or the only cause of climate change, whereas only 6% believe that climate change is mostly or entirely caused by natural factors, and 29% believe it is a mixture of both (Ipsos, 2018).

We addressed the following three competing hypotheses arising from the aforementioned ways in which text easiness and prior beliefs might affect claim judgment. The belief-consistency hypothesis expects in line with extant research (e.g., Lord et al., 1979; Kunda, 1990; Munro, 2010) that the influence of belief-consistency on claim evaluation outshines the easiness effect. Hence, after reading a belief-consistent text compared to a belief-inconsistent text, participants should agree more with the text’s claim, consider the source to be more credible, trust more in their own judgment and be less eager to consult an expert for judgment support, regardless of text easiness.

The differential effects hypothesis, in contrast, assumes analogous to the findings by Goldberg and Carmichael (2017) that both text easiness and belief consistency affect claim judgment, with belief consistency determining the direction of this judgment and easiness determining judgment strength and confidence. As a result, participants should agree more with belief-consistent than belief-consistent texts and rate the source of a belief-consistent text to be more credible. Their positive evaluation of a belief-consistent text/source and their negative evaluation of a belief-inconsistent text/source should be more pronounced if the text is easy rather than difficult. In addition, participants should trust more in their own judgment and be less eager to consult an expert after reading easy compared to difficult texts, regardless of belief consistency.

Finally, the moderation hypothesis, suggested by findings from Scharrer et al. (2013), assumes that text easiness affects the evaluation of only belief-consistent but not belief-inconsistent claims. Therefore, for belief-consistent but not for belief-inconsistent texts, agreement with the claim should be stronger and the evaluation of source credibility more positive after reading an easy compared to a difficult text. Participants should also trust more in their claim judgment and be less inclined to consult an expert after reading easy compared to difficult texts, but, again, these differences should occur only for belief-consistent information.

In addition to investigating these hypotheses, the present study explored what reasons participants provide for justifying their claim evaluation. Specifically, we sought to find out whether they refer explicitly to text easiness and prior beliefs as the reasons for their judgment in an open communication task.

## Materials and Methods

### Participants and Design

We sought to detect at least midsize effects, because previous research has shown effects of text easiness on participants’ claim evaluation to be of medium to large size (e.g., Scharrer et al., 2012, 2019). An *a priori* power analysis (Faul et al., 2007) revealed that a sample size of at least 36 participants was required to detect midsize effects with an alpha error probability of .05, and a power of .95. Based on this calculation, we aimed for a final sample of about 40 participants. To ensure the effectiveness of our belief-consistency manipulation, only participants who fulfilled two conditions were included in the final sample: First, they had to strongly believe in human behavior as the major cause of the current climate change [i.e., on a scale ranging from 1 (strong disbelief) to 7 (strong belief), they achieved a mean score of at least 5; see measures section below for a description of the prior beliefs scales]; second, they had to strongly disbelieve in non-human natural causes as the primary cause (i.e., on the respective scale they achieved a mean score of no more than 3). To reach the intended sample size, we collected data from 66 undergraduates (77% female, 23% male; *M* = 23.39 years, *SD* = 2.66) studying different majors at various German universities. Students from geology, geography, biology, meteorology, chemistry, and related sciences were excluded from participation to ensure participants’ non-expert status regarding the issue of climate change. Nineteen participants were excluded from further analyses because they did not fulfill the two predefined conditions, and a further four participants had to be excluded due to technical difficulties. The final sample contained 43 participants (79% female, 21% male; *M* = 23.23 years, *SD* = 2.17). On average, participants were studying in their seventh semester (*M* = 6.47, *SD* = 3.41), believed strongly in human behavior as the driving force of climate change [*M* = 6.10, *SD* = 0.65 on a scale ranging from 1 (strong disbelief) to 7 (strong belief)], and believed only slightly in other natural causes [*M* = 1.92, *SD* = 0.63 on a scale ranging from 1 (strong disbelief) to 7 (strong belief)]. In addition, they ascribed to themselves an intermediate to good level of prior knowledge on the topic of climate change [*M* = 2.68, *SD* = 0.97 on a scale ranging from 1 (very good) to (5 poor)] and considered the topic of climate change to be highly relevant [*M* = 6.23, *SD* = 0.95, on a scale from 1 (not at all relevant) to 7 (very relevant)]. Participants were recruited via student groups on Facebook.

The study used a 2 × 2 repeated measurement design with the factors belief consistency (texts were consistent vs. inconsistent with participants’ prior beliefs about the causes of climate change) and text comprehensibility (texts were easy vs. difficult to comprehend). Each participant was subjected to all four experimental conditions and thus read four target texts in total, each addressing a different proposition related to climate change.

### Materials

#### Reading Task

Participants were presented with a framing scenario to contextualize their text reading (e.g., Scharrer et al., 2012): they were asked to help a fictitious friend with her essay on the causes of climate change. The friend was particularly interested in one particular controversially discussed factor that might be causing climate change. Participants were then confronted with a text allegedly obtained from the internet that discussed the factor in question, and they were asked to read the text in order to advise their friend.

#### Stimulus Texts

The stimulus texts were brief argumentative texts about the causes of the current climate change (mean length = 237.5 words, *SD* = 18.78; see Supplementary Material for the English translation of a text example; the complete text materials in the original German version can be retrieved here: http://dx.doi.org/10.23668/psycharchives.4926). Each text began with a general statement about whether the current climate change is caused by either human or non-human factors. This was followed by a causal claim (the target claim) specifying which factor is mainly responsible for climate change. The claim was then supported by an explanation of the underlying mechanisms. The stimulus texts addressed four different topics related to the causes of climate change, so that participants were presented with a different topic in each experimental condition.

Two of the texts proposed claims consistent with participants’ beliefs and two texts proposed belief-inconsistent claims. In their initial statement, belief-consistent texts stated that the current climate change is anthropogenic. The target claim of the text then proposed that one specific human-related factor was primarily responsible for climate change. Hence, one of the belief-consistent texts claimed that “one of the major causes of the current climate change is the increased emission of methane gas caused by humans”; the other claimed that “one of the major causes of the current climate change is human-caused wood clearing.” Belief-inconsistent texts first stated that climate change is generally due to natural causes unrelated to human activity. The target claim then proposed a specific natural factor as the primary cause of climate change. Hence, the texts claimed either that “one of the major causes of the current climate change is cyclic changes in solar activity” or that “one of the major causes of the current climate change is the constant change of the earth’s orbit around the sun.”

In addition to belief consistency, the texts varied in terms of their easiness. For each target claim, two text versions were created: an easy-to-comprehend version and a difficult-to-comprehend version. Comprehensibility was manipulated through the use of technical terms and abbreviations: Whereas the difficult texts contained a high number of technical terms and abbreviations, the easy texts translated these terms into words familiar to non-expert audiences. This manipulation affected only the claim-supporting information, whereas the general statement at the beginning of the text and the target claim were easy to comprehend in all conditions to ensure that the effectiveness of the belief-consistency manipulation was unaffected by text easiness.

Two pilot studies were conducted to ensure the effectiveness of the belief-consistency and comprehensibility manipulations. In a first study, 28 students of different majors (68% female, 32% male; *M* = 25.64 years, *SD* = 3.52) who strongly believed in anthropogenic climate change indicated how strongly they agreed with the isolated target claims. As expected, they agreed more with the two claims designed to be belief-consistent than with the two claims designed to be belief-inconsistent, *t*(27) = 10.40, *p* > 0.001. Mean agreement with each belief-inconsistent claim was below 3 on a Likert scale ranging from 1 (don’t agree at all) to 7 (strongly agree) (sun activity: *M* = 2.68, *SD* = 1.76, earth’s orbit: *M* = 2.14, *SD* = 1.24). Mean agreement with each belief-consistent claim was above 5 (methane gas: *M* = 5.61, *SD* = 1.20, wood clearing: *M* = 5.86, *SD* = 1.33).

In a second pilot study, 26 students of different majors (89% female, 11% male; *M* = 23.01 years, *SD* = 3.96) rated the full texts in terms of their comprehensibility on a Likert scale ranging from 1 (not at all comprehensible) to 7 (very comprehensible). This confirmed that both text versions of each claim (easy and difficult to comprehend) differed significantly in terms of their perceived comprehensibility, all *t*s(24) > 4.1. All effect sizes were large and comparable between the belief-consistent (methane gas: Cohen’s *d* = 2.23, wood clearing: Cohen’s *d* = 1.62) and belief-inconsistent topics (sun activity: Cohen’s *d* = 1.77, earth’s orbit: Cohen’s *d* = 2.67). In addition, the comprehensibility ratings of the comprehensible and incomprehensible versions, respectively, did not differ between belief-consistent and belief-inconsistent topics [comprehensible: *t*(24) = 0.30, ns; less comprehensible: *t*(24) = 1.02, ns]. This indicates that the comprehensibility manipulation was independent of the belief-consistency manipulation.

### Measures

#### Prior Beliefs About the Causes of Climate Change

Prior to reading the text materials, participants reported their beliefs about the causes of climate change by rating their agreement with eight statements on 7-point items ranging from 1 (don’t believe at all) to 7 (strongly believe). Four statements measured their beliefs about human behavior being the major cause of climate change (“The current climate change is primarily caused by humans”; “The present climate change is mainly caused by human behavior”; “Human behavior is the major cause of the currently observed climate change”; “Essentially, the current climate change is caused by humanity,” Cronbach’s alpha = 0.781), and four statements measured beliefs about non-human, natural causes as the major reason for climate change (“The current climate change can be attributed primarily to natural causes”; “Natural factors that are independent of human behavior are the major cause of the current climate change”; “The currently observed climate change is a result of natural causes that have nothing to do with humans”; “The climate currently changes due to natural fluctuations,” Cronbach’s alpha = 0.737). The arithmetic means across the respective four statements served as the score for each participant’s prior belief in human and non-human causes of climate change and were used as a screening variable.

#### Agreement With the Text Claim

Before and after reading, participants’ agreement with the text claim was assessed with a 7-point item ranging from 1 (I don’t agree at all) to 7 (I fully agree).

#### Author Credibility

Participants indicated how credible they found the text’s author to be on a 7-point item ranging from 1 (not at all credible) to 7 (very credible).

#### Reliance on One’s Claim Judgment

Participants’ reliance on their claim judgment was assessed by measuring their preference for three different judgment strategies on 7-point items ranging from 1 (don’t agree) to 7 (strongly agree) (Scharrer et al., 2013, 2014).

Strategy 1: Trust in one’s own judgment based on present knowledge. Agreement with the statement “Based on my present knowledge about the topic, I am confident about deciding whether it is correct that (claim statement inserted)” reflected strong reliance on one’s judgment ability.

Strategy 2: Trust in one’s own judgment based on further information. Agreement with the statement “I want to obtain further information about (topic) that I shall then use to decide myself whether it is correct that (claim statement inserted)” indicated an intermediate level of reliance.

Strategy 3: Desire to outsource the judgment to an expert. Agreement with the statement “I want to obtain information about experts in the field in order to identify a particularly competent and credible expert. I would then consult this expert and rely on their judgment as to whether it is correct that (claim statement inserted)” indicated low reliance on one’s ability.

#### Reasons for the Claim Judgment

After judging their agreement with the target claim post-reading, participants were asked to write a letter to their fictitious friend in which they were to provide reasons for their claim judgment. The letters were coded according to whether participants named (1) text comprehensibility and/or (2) their prior beliefs as reasons for their claim judgment. Each of these variables was coded in a dichotomous format (contained/not contained). Twenty-five per cent of the letters were coded by two independent raters: the first author and a student assistant. After ensuring interrater agreement for both variables (Cohen’s kappas > 0.78), the remaining letters were coded by the first author.

1. Text comprehensibility was coded as a reason for one’s claim judgment if the participant referred explicitly to the high or low comprehensibility of the text to justify why they agreed or disagreed with the claim—for example: “The article explains the matter in an easily understandable fashion and is well written.”

2. Prior beliefs was coded if the participant based their claim agreement on topic information not mentioned in the text, or if the participant stated explicitly that they agreed/disagreed with the claim because it was in line with/contradicted their prior knowledge/beliefs—for example: “This is in accordance with my knowledge about the topic.”

### Procedure

The study was conducted online using Enterprise Feedback Suite (EFS) Survey. Upon starting the questionnaire, participants first indicated their prior beliefs about the causes of the current climate change. They then provided their demographic data before rating their own prior knowledge about climate change and the relevance of the topic. Afterward, they indicated their agreement with the isolated target claims (pre-measurement) before being presented with the first framing scenario and stimulus text. After reading, they again indicated their agreement with the claim (post-measurement) and provided the reasons for their claim judgment. They then indicated their reliance on their claim judgment and rated the author’s credibility. This was repeated so that every participant read one stimulus text from each experimental condition. Alternating with the texts on climate change, each participant was also presented with three distracter texts and framing scenarios on the topics of vaccination and genetically modified food to disguise the purpose of the experiment. Hence, each participant read seven texts in total. To control possible influences of presentation order or text topic on our results, the order of exposure to experimental conditions was counterbalanced among participants. Across the sample, all experimental conditions appeared equally often at the first, third, fifth, and seventh position, with the three distracter texts being placed randomly at the second, fourth, and sixth position. In addition, across the sample, each topic was addressed equally often by an easy and a difficult text. Participants were then debriefed, thanked, and asked to provide their email address to receive a 6-euro gift voucher as compensation for their participation. Participation lasted about 30–40 min.

## Results

For the detected effects, we provide information on the observed power (OP). The OP is a direct function of the observed *p*-value and therefore does not add any new information. However, we report the OP nonetheless to give readers a direct estimate of how likely it is that effects of the observed magnitude can be detected in potential future studies using the same sample size (O’Keefe, 2007).

### Preparatory Analysis

We first tested whether the presentation order of stimulus texts had any impact on participants’ response patterns using repeated measures ANOVAs with the independent factor position of target text (1–4) for all dependent variables. This revealed a significant order effect on Strategy 1 indicating participants’ reliance on their claim judgment, *F*(3, 126) = 4.64, *p* = 0.004, η*_*p*_*^2^ = 0.100, OP = 0.88. Trust in one’s own judgment based on present knowledge was lower after reading Text 1 than after reading Texts 2 and 4 (both *p*s < 0.03). In addition, there was a significant order effect on Strategy 2, *F*(2.39, 100.45) = 6.75, *p* < 0.001, η*_*p*_*^2^ = 0.139, OP = 0.97. Trust in one’s own judgment based on further information was stronger after reading the first than after reading the remaining target texts (all *p*s < 0.048). Presumably, participants were at first a bit uncertain regarding the particular judgment task, therefore preferring to seek further information rather than basing their judgment only on their current knowledge. However, they gained some confidence after getting used to the experimental setting. The remaining order effects did not attain significance, all *F*s < 1.63, ns.

We also sought to clarify whether there were any topic effects between both belief-consistent and both belief-inconsistent texts, respectively. We computed *t*-tests for dependent groups on all dependent variables and found a topic effect between both belief-consistent texts in terms of participants’ inclination to rely on Strategy 2, *t*(42) = 2.47, *p* = 0.018, OP = 0.82. Participants were more inclined to look for further information to support their judgment after reading the text on wood clearing than after reading the text on methane gas. The remaining comparisons were not significant, all *t*s < 1.67, ns.

Whereas the detected position and topic effects increased error variance, they could not account for any effects of our dependent variables, given that order of exposure to the experimental conditions and assignment of topics to the easy and difficult condition were counterbalanced across the sample.

### Main Analysis

Repeated measures ANOVAs with the independent factors text comprehensibility (easy vs. difficult to understand) and belief consistency (consistent vs. inconsistent) tested the influence of both factors on our quantitative dependent measures. For agreement with the text claim, time of measurement (pre- vs. post-reading) served as an additional independent factor. For the unpooled means and standard deviations of the quantitative measures see Table 1.

**TABLE 1:** Means and standard deviations (in parentheses) for pre- and post-measures of claim agreement, preference of strategies indicating reliance on one’s claim judgment, and source credibility as a function of belief consistency and text comprehensibility.

		Consistent/easy	Consistent/difficult	Inconsistent/easy	Inconsistent/difficult
Claim agreement	Pre	5.58 (1.10)	5.30 (1.41)	2.63 (1.40)	2.88 (1.37)
	Post	5.63 (1.41)	4.79 (1.52)	3.16 (1.56)	2.95 (1.73)
Strategy 1		4.56 (1.80)	3.51 (1.79)	3.35 (1.85)	3.05 (1.99)
Strategy 2		5.02 (1.70)	5.63 (1.62)	5.30 (1.81)	5.33 (1.78)
Strategy 3		3.88 (1.84)	4.86 (1.81)	4.67 (1.74)	4.40 (1.83)
Source credibility		5.40 (1.45)	3.95 (1.75)	3.26 (1.51)	3.02 (1.54)

**Measurement scales ranged from 1 to 7, with high values indicating high agreement/strategy preference/credibility.**

#### Agreement With the Text Claim

There was a significant main effect of comprehensibility, *F*(1, 42) = 4.18, *p* = 0.047, η*_*p*_*^2^ = 0.090, OP = 0.52, due to participants agreeing more with the claim from an easy [estimated marginal mean (EMM) = 4.25, *SE* = 0.11] than from a difficult text (EMM = 3.98, *SE* = 0.14). In addition, agreement was higher for belief-consistent (EMM = 5.33, *SE* = 0.13) than for belief-inconsistent texts (EMM = 2.91, *SE* = 0.18), *F*(1, 42) = 117.10, *p* < 0.001, η*_*p*_*^2^ = 0.736, OP = 1.00. These main effects were qualified by significant interactions between comprehensibility and consistency, *F*(1, 42) = 5.84, *p* = 0.020, η*_*p*_*^2^ = 0.122, OP = 0.66, and between comprehensibility and time of measurement, *F*(1, 42) = 5.71, *p* = 0.021, η*_*p*_*^2^ = 0.120, OP = 0.65. No other effects attained significance, all Fs < 3.35, ns.

To follow up on these interactions, ANOVAs with the independent factors comprehensibility and belief consistency were calculated separately for pre- and post-measures of claim agreement. For the pre-measures, there was a significant main effect of belief consistency, *F*(1, 42) = 128.36, *p* < 0.001, η*_*p*_*^2^ = 0.753, OP = 1.00. Before reading, and in accordance with our belief-consistency manipulation, participants agreed more with belief-consistent (EMM = 5.44, *SE* = 0.15) than with belief-inconsistent claims (EMM = 2.76, *SE* = 0.19). No other effects attained significance, all Fs < 3.35, ns.

**FIGURE 1:**
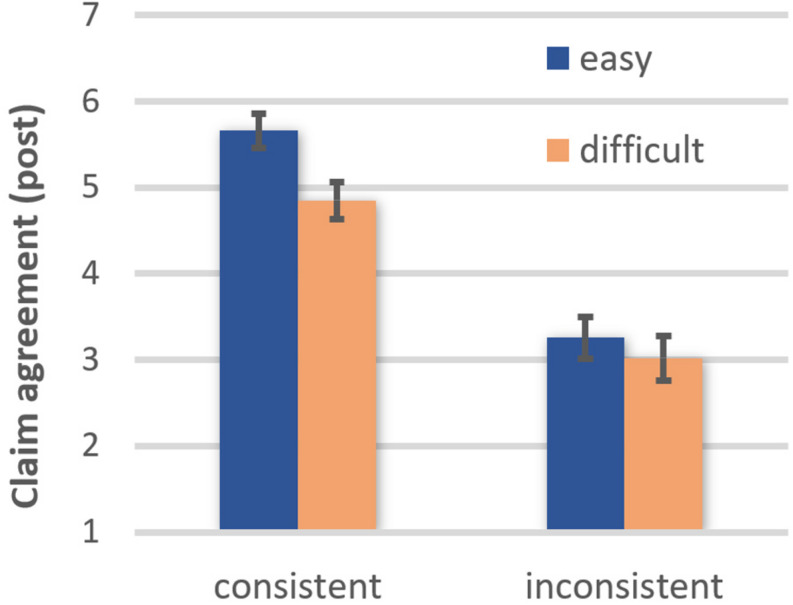
Effects of text comprehensibility on agreement with the text claim after reading as a function of belief-consistency (error bars represent standard errors).

For the post-measures, participants’ claim agreement was overall greater after reading an easy (EMM = 4.40, *SE* = 0.16) than a difficult text (EMM = 3.87, *SE* = 0.18), *F*(1, 42) = 7.44, *p* = 0.009, η*_*p*_*^2^ = 0.150, OP = 0.76. Moreover, participants agreed more with belief-consistent (EMM = 5.21, *SE* = 0.18) than with belief-inconsistent texts (EMM = 3.06, *SE* = 0.22), *F*(1, 42) = 53.48, *p* < 0.001, η*_*p*_*^2^ = 0.560, OP = 1.00. These main effects were qualified by a marginally significant comprehensibility × belief consistency interaction, *F*(1, 42) = 3.56, *p* = 0.066, ηp2 = 0.078, OP = 0.45 (see Figure 1). Follow-up *t*-tests showed that agreement was greater after reading an easy than a difficult belief-consistent text, *t*(42) = 3.25, *p* = 0.002, OP = 0.94. In contrast, comprehensibility had no impact on agreement with belief-inconsistent texts, *t*(42) = 0.84, ns. These results are consistent with the moderation hypothesis positing that belief consistency moderates the easiness effect on claim agreement.

#### Author Credibility

**FIGURE 2:**
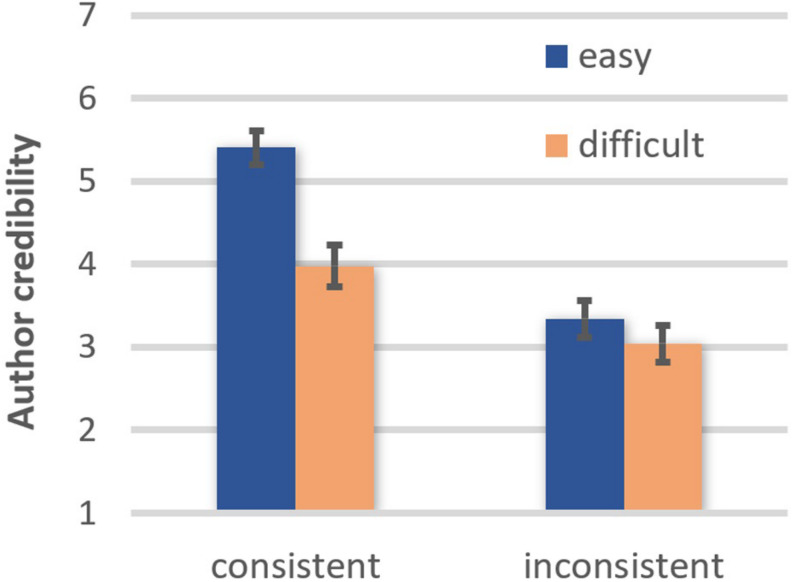
Effects of text comprehensibility on author credibility as a function of belief-consistency (error bars represent standard errors).

The author of an easy text (EMM = 4.33, *SE* = 0.17) was perceived to be more credible than the author of a difficult text (EMM = 3.49, *SE* = 0.21), *F*(1, 42) = 19.08, *p* < 0.001, η*_*p*_*^2^ = 0.312, OP = 0.99. In addition, the author of a belief-consistent text (EMM = 4.67, *SE* = 0.17) was perceived to be more credible than the author of a belief-inconsistent text (EMM = 3.14, *SE* = 0.19), *F*(1, 42) = 49.36, *p* < 0.001, η*_*p*_*^2^ = 0.540, OP = 1.00. Furthermore, there was a comprehensibility × belief-consistency interaction, *F*(1, 42) = 9.74, *p* = 0.003, η*_*p*_*^2^ = 0.188, OP = 0.86 (see Figure 2). The author of a belief-consistent easy text was perceived to be more credible than the author of a belief-consistent difficult text, *t*(42) = 5.32, *p* < 0.001, OP = 1.00. In contrast, comprehensibility had no impact on how credible the author of a belief-inconsistent text was perceived to be, *t*(42) = 0.85, ns. This pattern of results is consistent with the moderation hypothesis.

#### Reliance on One’s Claim Judgment

**FIGURE 3:**
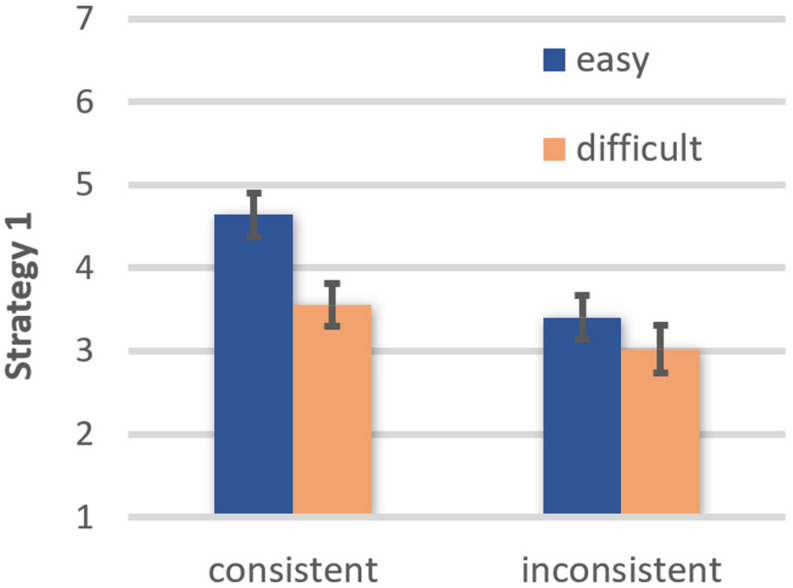
Effects of text comprehensibility on trust in one’s own judgment based on present knowledge (Strategy 1) as a function of belief-consistency (error bars represent standard errors).

Strategy 1. A main effect of comprehensibility, *F*(1, 42) = 7.34, *p* = 0.010, η*_*p*_*^2^ = 0.149, OP = 0.75, showed that participants trusted more in their judgment based on their present knowledge after reading easy (EMM = 3.95, *SE* = 0.25) than difficult texts (EMM = 3.28, *SE* = 0.25). They also trusted more in their judgment after reading belief-consistent (EMM = 4.04, *SE* = 0.22) than belief-inconsistent texts (EMM = 3.20, *SE* = 0.26), *F*(1, 42) = 12.87, *p* = 0.001, η*_*p*_*^2^ = 0.235, OP = 0.94. Furthermore, a significant comprehensibility × belief consistency interaction, *F*(1, 42) = 4.95, *p* = 0.032, η*_*p*_*^2^ = 0.105, OP = 0.58, revealed that for belief-consistent texts, trust was higher if the text was easy than difficult to understand, *t*(42) = 3.19, *p* = 0.003, OP = 0.93. In contrast, and consistent with the moderation hypothesis, comprehensibility had no impact on participants’ trust in their own judgment based on present knowledge when reading belief-inconsistent texts, *t*(42) = 1.12, ns (see Figure 3).

**FIGURE 4:**
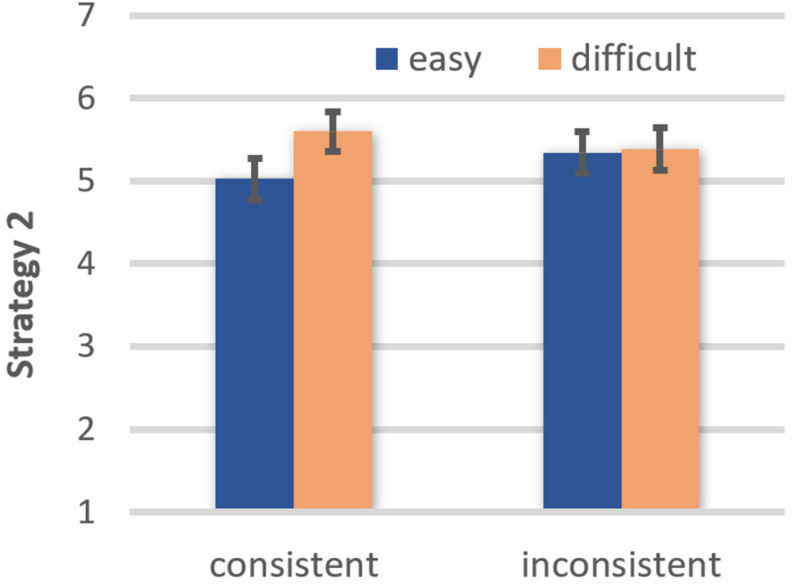
Effects of text comprehensibility on trust in one’s own judgment based on further information (Strategy 2) as a function of belief-consistency (error bars represent standard errors).

Strategy 2. There was a main effect of comprehensibility, *F*(1, 42) = 4.14, *p* = 0.048, η*_*p*_*^2^ = 0.090, OP = 0.51, due to participants being less inclined to trust in their judgment based on further information after reading easy (EMM = 5.16, *SE* = 0.22) than difficult texts (EMM = 5.48, *SE* = 0.23). Although the main effect of belief consistency did not attain significance, *F*(1, 42) = 0.003, ns, there was a marginally significant comprehensibility × belief consistency interaction, *F*(1, 42) = 3.95, *p* = 0.053, η*_*p*_*^2^ = 0.086, OP = 0.49 (see Figure 4). For belief-consistent texts, trust in one’s own decision based on further information was higher when the text was easy rather than difficult to understand, *t*(42) = 2.47, *p* = 0.018, OP = 0.79. However, comprehensibility had no impact on the popularity of Strategy 2 when reading belief-inconsistent texts, *t*(42) = 0.13, ns.

**FIGURE 5:**
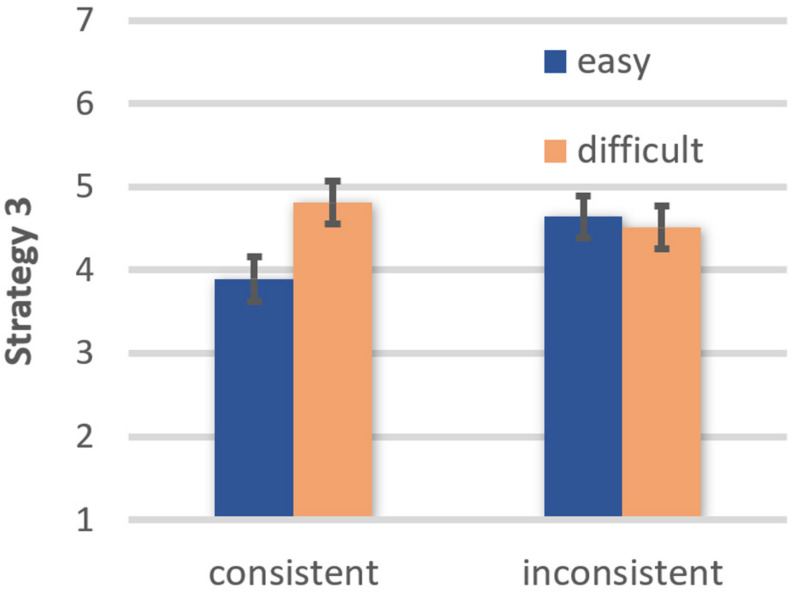
Effects of text comprehensibility on desire to outsource the judgment to an expert (Strategy 3) as a function of belief-consistency (error bars represent standard errors).

Strategy 3. Participants were more inclined to seek advice from an expert after reading easy (EMM = 4.28, *SE* = 0.24) than difficult texts (EMM = 4.63, *SE* = 0.25), *F*(1, 42) = 6.81, *p* = 0.013, η*_*p*_*^2^ = 0.140, OP = 0.72. There was no main effect of belief consistency, *F*(1, 42) = 0.83, ns, but a significant comprehensibility × belief consistency interaction, *F*(1, 42) = 16.35, *p* < 0.001, η*_*p*_*^2^ = 0.280, OP = 0.98 (see Figure 5). Consistent with the moderation hypothesis, the desire to consult an expert was stronger after reading a belief-consistent easy than difficult text, *t*(42) = 4.67, *p* < 0.001, OP = 1.00. In contrast, comprehensibility had no impact on the desire for expert advice if texts were belief-inconsistent, *t*(42) = 1.39, ns.

#### Reasons for the Claim Judgment

1. Text comprehensibility as a reason for one’s claim judgment. Descriptive statistics showed that about one third of participants justifying their claim judgment referred to (a lack of) comprehensibility if they had read a difficult text. In contrast, only a few participants named comprehensibility as a reason for their claim judgment if texts were easy (Table 2). A Cochran *Q*-test showed that this difference was significant, Q(3, *N* = 43) = 17.18, *p* = 0.001.

**TABLE 2:** Relative frequencies of reasons given for one’s claim judgment as a function of belief consistency and text comprehensibility.

	Consistent/easy	Consistent/difficult	Inconsistent/easy	Inconsistent/difficult
Text comprehensibility as a reason for claim judgment	14%	35%	7%	37%
Prior beliefs as a reason for claim judgment	51%	42%	51%	49%

2. Prior beliefs as a reason for one’s claim judgment. Participants frequently referred to prior beliefs to justify their claim judgment. They did so in about 50% of cases across conditions (Table 2), Q(3, *N* = 43) = 1.42, ns.

## Discussion

The present results show that undergraduates who believe in anthropogenic climate-change are affected by text easiness when evaluating belief-related scientific claims—but only if these claims are in accordance with their beliefs. These findings are consistent with the moderation hypothesis proposing that text easiness affects non-experts’ evaluation of only belief-consistent information. After reading belief-consistent information, our participants agreed more with claims from easy than difficult texts and found the source of easy texts to be more credible. In contrast, participants were reluctant to agree with the claim and to deem the source credible if the text was inconsistent with their beliefs, regardless of text easiness. Apparently, our participants felt sufficiently confident to strongly agree with a claim that was consistent with their beliefs and that was supported by information they seemed well able to understand. However, if this supporting information was difficult to understand, participants became more hesitant in their judgment of claim and source. A similar hesitancy also seems to have been induced by belief-inconsistent information.

This interpretation is in accordance with the results on participants’ reliance on their own claim judgment. If information was consistent with their beliefs and easy to understand, participants trusted in their own claim judgment quite strongly and were not much inclined to consult an expert for judgment support. This reliance on their own judgment was reduced when participants encountered belief-consistent information that was difficult to understand. This was in line with the easiness effect previously observed in the evaluation of claims about which non-experts hold no prior belief (e.g., Scharrer et al., 2012). However, if information was inconsistent with their prior beliefs, participants were generally insecure in their evaluation, expressed reduced trust in their own judgment, and reported a relatively increased desire to consult an expert. This decreased reliance on their own judgment abilities was again independent of information easiness.

The apparent relevance that participants attached to both belief consistency and text easiness as evaluation criteria is also reflected in the explicit reasons they provided for their claim judgment. Across conditions, belief consistency was named by about one half of the participants as a reason for their judgment. Text comprehensibility was mentioned rather often as well (in about one third of cases), but notably only if texts were difficult to comprehend. Apparently, our participants took it for granted that scientific texts should be easy to understand and only considered it remarkable and relevant for their validity judgment when texts did not meet this expectation.

These findings are in line with previous research on the impact of text easiness, suggesting that non-experts attach weight to their own perceived understanding of the subject matter when judging a scientific claim in spite of their lack of knowledge (e.g., Scharrer et al., 2012, 2017; Thon and Jucks, 2017; Bullock et al., 2019). However, the present results also suggest that, at least for undergraduates who believe in anthropogenic climate-change, text easiness loses its influence when information is inconsistent with their prior beliefs. One explanation for this finding is that non-experts are so skeptical toward belief-inconsistent information that they firmly reject it without feeling the need to pay any attention to other information features such as easiness. However, the descriptive statistics for participants’ reliance on their own judgment appear to contradict the notion that belief-inconsistent claims were discounted confidently. Instead, they suggest that the increased confidence triggered by text easiness is prevented when information is inconsistent with prior beliefs. It seems that encountering information that challenges previous beliefs increased participants’ awareness of their own epistemic limitations. These findings are in accordance with previous research showing that the seductive effect of text easiness can be mitigated through inducing epistemic doubt among non-experts by making them aware that the claim in question is discussed controversially among sources (Scharrer et al., 2013) or by warning them that the topic is, in fact, very complex (Scharrer et al., 2014). The present results add to this research by suggesting that encountering controversy between an external source and one’s own prior beliefs might create a similar kind of doubt that impedes the easiness effect.

### Limitations and Future Directions

It should be noted that based on previous research on the easiness effect (e.g., Scharrer et al., 2012, 2019), the present sample size was optimized to detect effects of at least medium size—and the effects of text easiness presently obtained at the level of belief-consistent texts were indeed medium- to large-sized. Due to the chosen sample-size, we cannot conclude with certainty that belief-inconsistency prevented the influence of text easiness altogether. It is possible that an easiness effect still occurred even if a text was inconsistent with participants’ prior beliefs, albeit with an effect size too small to be detected in our sample. Nonetheless, such a decreased rather than eliminated easiness effect would still be consistent with the moderation hypothesis.

In addition, the present study focused only on individuals who assented to the claim that human behavior is the primary cause of climate change. This is the most prevalent attitude among the German public (Ipsos, 2018); nonetheless, to test the generalizability of our results to people holding different positions, future research should also focus on individuals who reject this claim or who believe that both human and non-human factors contribute to climate change.

Another limitation of the present study is that the stimulus materials focused on only the topic of climate change. This topic has some specific properties that set it apart from many other scientific issues: Climate change is subject to intense public debate, and famously, there is broad expert consensus on human-caused global warming. Both factors might have enhanced our participants’ confidence in and their adherence to their prior beliefs. It is possible that non-experts attribute less weight to their prior beliefs when engaging with less debated topics and/or topics on which expert opinion is less clear. Future research should examine whether the present results generalize to the evaluation of claims about other issues.

Finally, future research should also investigate how far the present results are applicable to non-experts from other educational backgrounds. The present study focused on undergraduate students who, despite not being experts on climate change, had some experience with academic methods and research in general. Whereas Scharrer et al. (2017) showed that the easiness effect can be observed among a more heterogeneous sample consisting of non-experts with and without an academic background, it is conceivable that the inclination to overestimate one’s own evaluative capabilities might be even higher in a purely non-academic sample. Individuals unfamiliar with science and research may be less aware of the generally high complexity of scientific knowledge. Impressions of their own evaluative capabilities might therefore be influenced even more by the easiness of encountered text information. The resulting increase in the strength of the easiness effect could then even override the mitigating influence of belief inconsistency.

### Educational Implications

The finding that participants overlooked their own epistemic limitations when evaluating easily comprehensible belief-consistent information suggests that information providers can confirm or even more deeply entrench previously held beliefs by presenting a simple text. One could argue that the present results are not all that problematic, given that the belief-consistent information in our study argued in favor of anthropogenic climate change and therefore reflects the scientific consensus. Hence, it might be considered more important (and fortunate) that text easiness did not make our participants embrace the belief-inconsistent and thus scientifically untenable point of view. However, non-experts’ prior beliefs are, of course, not always scientifically correct. It is concerning that misconceptions might be further strengthened by easy accounts, especially given that pseudoscience and conspiracy theories are often characterized by simple explanations and depictions, and that the sense of understanding which is fostered by this simplicity is deliberately interpreted as an indication of truth (Boudry et al., 2015; Bromme, 2020).

If the presently obtained effects generalize to other science topics and to non-experts from different educational backgrounds holding different prior beliefs, various implications for educational practice would arise. Educational measures could help to reduce non-experts’ vulnerability to misinformation by better preparing them for their consumption of simplified science accounts and immunizing against the seductive effect of text easiness (see also Reber and Greifeneder, 2017). Non-experts need to recognize that, due to the division of cognitive labor in our societies, they have to approach such accounts as competent outsiders. This implies relying on evaluation criteria that account for non-expert’s epistemic limitations, such as source evaluation and corroboration, rather than evaluating contents directly using inadequate criteria such their subjective feeling of understanding (Feinstein, 2011; Bromme and Goldman, 2014; Duncan et al., 2018).

Such educational measures could build on the presently observed mitigating influence of belief inconsistency. By presenting science students with information that counters their previous misconceptions, it may be possible to induce general doubt about their own epistemic capabilities. This could be used as a starting point to raise awareness of the complex nature of scientific knowledge and the wealth of background knowledge required for truly informed validity judgments.

Students could also be taught directly about the inadequacy of easiness as a cue for validity of scientific information. Duncan et al. (2018) suggest that non-experts’ uncritical reliance on possibly oversimplified accounts might be reduced by familiarizing them with the norms of scientific discourse, such as the importance of technical terms for precise communication and the frequent hedging of claims that reflect the probabilistic nature of scientific findings. By also teaching students about the aims of (journalistic) science simplification and the means by which these aims are often achieved, such as removal of critical details and hedging, educators may foster awareness that simplified accounts might promote imprecise or even inaccurate conclusions (Duncan et al., 2018).

## Data Availability Statement

The data presented in the study are deposited in the PsychArchives repository, accession number: doi: 10.23668/psycharchives.4927.

## Ethics Statement

Ethical review and approval was not required for the study on human participants in accordance with the local legislation and institutional requirements. The patients/participants provided their written informed consent to participate in this study.

## Author Contributions

LS, MS, and RB conceived the experimental idea. LS planned and conducted the experiment and performed data analysis. LS prepared the manuscript, which was subsequently critically reviewed by all authors.

## Conflict of Interest

The authors declare that the research was conducted in the absence of any commercial or financial relationships that could be construed as a potential conflict of interest.

## Publisher’s Note

All claims expressed in this article are solely those of the authors and do not necessarily represent those of their affiliated organizations, or those of the publisher, the editors and the reviewers. Any product that may be evaluated in this article, or claim that may be made by its manufacturer, is not guaranteed or endorsed by the publisher.
