# Supplementary material for: Information Easiness Affects Non-experts’ Evaluation of Scientific Claims About Which They Hold Prior Beliefs
Source: Front Psychol. 2021 Aug 27;12:678313. doi: 10.3389/fpsyg.2021.678313 (PMC8430255; doi:10.3389/fpsyg.2021.678313)
Supplement: Supplementary file 1 [file Data_Sheet_1.PDF]

Supplementary Material

Example of the easy- and difficult-to-comprehend version of a belief-inconsistent stimulus text

Belief-inconsistent, easy to comprehend

It is a widespread belief that the current climate change is predominantly caused by human activity. However, from a scientific point of view, this position is not tenable. In fact, it is primarily natural causes that are responsible for the change in climate.

One of the major causes of the current climate change is the constant change of the earth's orbit around the sun. This is because the earth's orbit around the sun changes its shape over the course of several hundred years (its so-called 'eccentricity"), and it shifts from almost round to about five per cent elliptic. The major cause of this variation is the gravitational pull of the planets Venus and the massive Jupiter, and, to a lesser extent, that of other planets in our solar system. Depending on their position, they attract the earth sometimes to a stronger, sometimes to a weaker extent.

Because the shape of the earth's orbit influences the irradiation of sun energy, every change in the orbit's shape brings about a change in the amount of radiation reaching the earth's surface. An earth's orbit that has an almost round shape means low eccentricity and less radiation, whereas an elliptic shape means high eccentricity and much radiation. This is because in the event of an elliptic orbit, the earth is, on average, closer to the sun. Currently, the earth is circling around the sun in an increasingly elliptic shape. These changes towards an elliptic shape contribute to rising temperatures on earth.

Apart from the changes in the earth's orbit, further natural, nonhuman causes of the current climate change also need to be considered.

Belief-inconsistent, difficult to comprehend

It is a widespread belief that the current climate change is predominantly caused by human activity. However, from a scientific point of view, this position is not tenable. In fact, it is primarily natural causes that are responsible for the change in climate.

One of the major causes of the current climate change is the constant change of the earth's orbit around the sun. This is because the revolution of PE around the central body changes its eccentricity over the course of several hundred years. In doing so, the orbit shifts from a numerical eccentricity of 0.0006 to an eccentricity of 0.058. The major cause of this variation is the FG of Veneris and the

massive Lovis, and, to a lesser extent, that of other DP and GP. Depending on their position, they exert a sometimes stronger, sometimes weaker FG on PE.

Because the shape of the body’s revolution influences the irradiation of SE, every change in the orbit’s shape brings about a change in the amount of radiation reaching the PES. Low eccentricity means less radiation, whereas high eccentricity means much radiation. This is because in the event of a Kepler orbit, PE, averaged across perihel and aphel, is closer to the central body. Currently, PE circles around sol with increasingly high eccentricity. These changes towards a Kepler shape contribute to rising temperatures on earth.

Apart from the changes in the earth’s orbit, further natural, nonhuman causes of the current climate change also need to be considered.
